# A Review of the Nursery Culture of Mud Crabs, Genus *Scylla*: Current Progress and Future Directions

**DOI:** 10.3390/ani11072034

**Published:** 2021-07-08

**Authors:** Muhammad Nur Syafaat, Mohamad Nor Azra, Khor Waiho, Hanafiah Fazhan, Ambok Bolong Abol-Munafi, Sairatul Dahlianis Ishak, Mohammad Syahnon, Azmie Ghazali, Hongyu Ma, Mhd Ikhwanuddin

**Affiliations:** 1 Research Institute for Brackishwater Aquaculture and Fisheries Extension, Maros 90512, Indonesia; muhammad.nursyafaat@kkp.go.id; 2 Higher Institution Centre of Excellence (HICoE), Institute of Tropical Aquaculture and Fisheries, Universiti Malaysia Terengganu, Kuala Nerus 21030, Malaysia; waiho@umt.edu.my (K.W.); fazhan@umt.edu.my (H.F.); munafi@umt.edu.my (A.B.A.-M.); sairatul.ishak@umt.edu.my (S.D.I.); syahnon.m@umt.edu.my (M.S.); azmie@umt.edu.my (A.G.); 3 Institute for Tropical Biodiversity and Sustainable Development (IBTPL), Universiti Malaysia Terengganu, Kuala Nerus 21030, Malaysia; azramn@umt.edu.my; 4 STU-UMT Joint Shellfish Research Laboratory, Shantou University, Shantou 515063, China; mahy@stu.edu.cn; 5 Guangdong Provincial Key Laboratory of Marine Biotechnology, Shantou University, Shantou 515063, China; 6 Faculty of Fisheries and Marine, Campus C, Airlangga University, Mulyorejo, Surabaya 60115, Indonesia

**Keywords:** aquaculture, shellfish, future food, nursery stage, mud crab, juvenile, seed production, hatchery, crablet, breeding

## Abstract

**Simple Summary:**

Nursery culture knowledge is important for successful commercial seed production especially for mud crab, genus *Scylla*, a highly valued delicacy. The aim of this review paper is to summarise the status and information on the current nursery culture stage of the mud crab. Various aspects of mud crab hatchery described in this paper are expected to facilitate practitioners and lay people to easily understand a mud crab nursery. This review also provides guidelines for researchers in conducting future research and development on a mud crab nursery in order to increase the production of mud crab crablets for the farming industry.

**Abstract:**

The nursery stages of mud crab, genus *Scylla*, proceed from the megalopa stage to crablet instar stages. We review the definition and several of the key stages in mud crab nursery activities. The practice of the direct stocking of megalopa into ponds is not recommended due to their sensitivity. Instead, nursery rearing is needed to grow-out mud crabs of a larger size before pond stocking. Individual nursery rearing results in a higher survival rate at the expense of growth and a more complicated maintenance process compared with communal rearing. The nursery of mud crabs can be done both indoors or outdoors with adequate shelter and feed required to obtain a good survival percentage and growth performance. *Artemia* nauplii are still irreplaceable as nursery feed, particularly at the megalopa stage, while the survival rate may be improved if live feed is combined with artificial feed such as microbound diet formulations. Water quality parameters, identical to those proposed in tiger shrimp cultures, can be implemented in mud crab rearing. The transportation of crablets between different locations can be done with or without water. The provision of monosex seeds from mud crab hatcheries is expected to become commonplace, increasing seed price and thus improving the income of farmers. Numerous aspects of a mud crab nursery including nutrition; feeding strategies; understanding their behaviour, i.e., cannibalism; control of environmental factors and practical rearing techniques still need further improvement.

## 1. Introduction

Mud crabs of the genus *Scylla* are a seafood product that sees high demand in both local and international markets [1,2]. Currently, there are four known species in the genus *Scylla*: *S. olivacea*, *S. paramamosain*, *S. serrata* and *S. tranquebarica*. All species are reported to have been commercially farmed [2,3]. The demand for mud crab *Scylla* increases annually [3], which has led to overfishing as most mud crab landings still rely heavily on captured fisheries [4,5]. The impact on wild populations could be lessened if sufficient support was received from the aquaculture sector in the form of seed supply from hatcheries [2,6,7]. In addition, mud crab grow-out and fattening activities incorporated within the mangrove area—known as silvo-fishery techniques [8,9,10], culture in earthen ponds [11,12] or in concrete ponds [13] and grow-out activity in indoor conditions [14,15]—could all contribute to the hatchery replacement of a wild catch. However, mud crab hatchery activities continue to be constrained by low survival rates; hence, the supply of seeds is still very limited for large-scale aquaculture purposes [2,16]. Disease control and the improved nutritional condition of larvae in seed production tanks also need to be developed for the reliable mass seed production of portunid crabs to be implemented [17].

**Figure 1:**
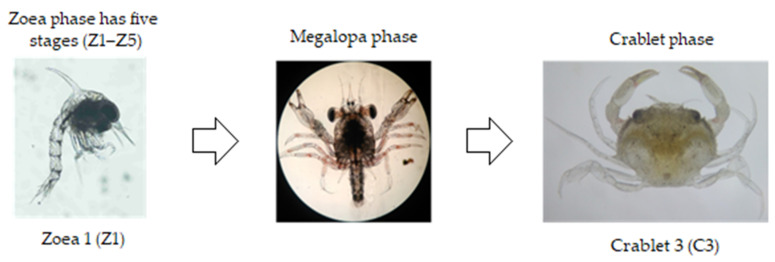
Larvae development of mud crabs from zoea to crablet stages.

Mud crab hatcheries typically involve the maintenance of larvae to crablet size (>5 mm) before being marketed to farmers [12,18]. The maintenance of mud crab larvae in the hatchery has three stages: the Zoea 1 (Z1) to Zoea 5 (Z5) larvae, Z5 to Megalopa (M) and the maintenance of M to the crablet phase [19] (Figure 1). The hatchery production process of mud crab crablet takes about 30 days (starting from Z1 to Crablet 2–3 (C2–C3)) [20]. Unlike *Portunus pelagicus*, where a high survival has been observed when stocking into ponds at the megalopae phase, the critical transition step of M to Crablet 1 (C1) in mud crabs has produced inconsistent and unreliable results, with high mortality often reported during the metamorphosis from M to the C1 stage [21,22]. Therefore, the nursery phase of mud crabs (from megalopae to early crablet stages (≥C3)) needs to be improved for a large number of high quality crablets [17,23,24]. The larval rearing process was thoroughly reviewed by Waiho et al. [2] while the current review focuses on rearing techniques and parameters involved in the nursery phase of mud crab, genus *Scylla*, including the principal technical practices of a mud crab nursery from stocking to transportation. Several issues during this phase are identified and potential solutions are discussed.

## 2. Mud Crab Nursery

The nursery stage of mud crab, genus *Scylla*, includes one megalopa stage and subsequent crab instar stages. The transition from M into the C1 stage is around 6–7 days [3,22] whereas the duration (moulting interval) from C1 to subsequent crablet stages increase gradually as the stages increase [25] (Table 1). The maintenance of the M stage to the next C1 stage is identified as a critical period during mud crab larvae rearing. Among the assumed bottlenecks of this phase are (i) nutritional factors that trigger the occurrence of Moulting Death Syndrome (MDS) [26] and (ii) a high level of cannibalism as M is the stage when pincers appear [27,28,29]. The maintenance of M to C1 of mud crabs requires serious care because the survival rate remains inconsistent and very low (<50%). Studies related to nutritional, environmental and behavioural aspects at this phase are needed to optimise the seed production from hatcheries. Aside from M to the crablet phase, the extended nursery stage (started from a certain crablet phase to a larger crablet phase) of mud crabs is done to produce a larger size of young crabs [28,30,31]. Generally, a mud crab nursery lasts for 2–4 weeks [3,22,27,28,29]. The current practices of existing nurseries are listed in the following section.

## 3. Mud Crab Nursery Techniques

### 3.1. Nursery Area

The nursery area may be located indoors or outdoors. M are nursed in concrete, fiberglass or plastic tanks or net cages within brackish water ponds (Table 2) [22,23,27,31,32]. The nursery of mud crabs in indoor conditions allows for a better control of various parameters, e.g., water quality, water exchange, siphoning of waste and excess feed, as well as improving access for the daily observation of crablets. However, the production and growth of crablets in net cages or in ponds has shown an improved physical performance and an increased survival [31]. Natural lighting accompanied by the presence of natural food in ponds are believed to be the trigger factors for the growth of crablets maintained in net cages.

The nursery of mud crabs in indoor conditions still needs to be further investigated in order to optimise the hatchery production of crablets. Among the methods that can be applied to indoor mud crab protocols is biofloc technology. Further research is needed to determine whether the biofloc system used in the shrimp farming industry can be used for future M maintenance and whether feed in the form of a biofloc meal can be a food substitution for M and crablets.

### 3.2. Rearing Techniques

Another important consideration is whether to rear individually or communally. The nursery rearing of M individually (indoor condition) in good water quality conditions could raise the survival up to 90–100% [24,33]. Although a high survival rate is valuable, extra effort is required to implement the individual crablet rearing technique especially during feeding. Automatic feed machines during individual M rearing could be developed to have low capital and operating costs and to reduce high manpower costs and the inefficiency of manual feeding.

In the communal rearing technique, cannibalism is a major problem. To reduce cannibalism, black nylon, bunched netting or seaweed may be placed at the bottom of the rearing tanks as shelters while a few may also be allowed to float in the water column [22,31,34,35]. During the crablet or juvenile stages, shelter options include plastic strings, bamboo tubes and an open sand substratum [34] or brick and shell shelters [28]. The presence of shelters and the availability of sufficient feed greatly reduce the rate of cannibalism [36], which is reasonable as decapod larvae usually associate with floating leaves and clumps of algae in natural conditions and this behaviour could reduce predation, save energy when close to the turbid water surface or function as a transport mechanism [37]. Studies related to the nursery of mud crabs started at megalopa and crablet stages with a different treatment and the effect on the survival rate is summarised in Table 2.

**Table 2:** Summary of the studies related to the nursery of mud crabs (*Scylla* spp.) starting at megalopa and crablet stages with a different treatment (stocking density, rearing medium, type of shelter or feeding regimes) and the effect on the survival rate.

Initial Stage of Mud Crab (*Scylla* spp.)	Treatment	Stocking Density	Rearing Medium	Type of Shelter	Feeding	Rearing Duration (Days)	SR (%)	SGR-_CW_ (%)	Reference
M to C1 (*S. serrata*)	Feeding regimes: (a) *Artemia* nauplii, (b) boosted *Artemia*, (c) dried shrimp (*Acetes* spp.), (d) dried mud worm (*Marphysa* spp.)	10 ind/L	Bowl of 5 L volume (filled 3 L culture water)	none	(a) (b) (c) (d) (a,b) (a,c) (a,d) (b,c) (b,d) (c,d)	Until M metamorphosed to crabs or died	38.9 46.7 12.8 6.7 49.4 40.0 57.8 45.6 41.7 6.1		[16]
Z5 and M (mix) to C2–3 (*S. serrata*)	Feeding regimes	133 ind/m^2^	Aquarium	Paranet pieces	*Artemia* nauplii only *Artemia* nauplii + PL (post larvae) shrimp feed *Artemia* nauplii + dried shrimp *Artemia* nauplii + shrimp feed	14	26.66 31.66 20.00 11.66		[19]
M to C1 (*Scylla* spp.)	Salinity 24–30 ppt Temperature 25–30 °C pH 7.5–8.5 Water depth 60–80 cm	30 ind/m^2^	20 m^2^ (4 × 5 m) net cages	Bunched black nets and coconut fronds	Phytoplankton and zooplankton grown with organic and inorganic fertilisers	30	30–50		[22]
M to crablet stages (*S. serrata*)	Stocking density	10 ind/m^2^ 20 ind/m^2^ 30 ind/m^2^	Net cages	Dried coconut fronds	Macerated brown mussel meat (*Modiolus metcalfei)* or fish (20–30% of biomass)	30	53.33 48.30 50.00		[27]
M to crablet stages (*S. serrata*)	Salinity 26 ppt Salinity 32 ppt	1000 ind/m^3^	Concrete tanks and net cages	PVC pipe cuttings, black nets, seaweed *Gracilariopsis bailinae*	*Artemia* nauplii, adult artemia, trash fish, green mussel or *Acetes*	18	40.10 26.20		[38]
M to C1 (*S. serrata*)	Feeding regimes	12 ind/L	Tall conical-based plastic	None	100% MBD 100% *Artemia* 75% MBD:25% *Artemia* 50% MBD:50% *Artemia* 25% MBD:75% *Artemia*	Until M metamorphosed to C1or died	±6.5 ±5.0 ±5.0 ±3.5 ±8.5		[39]
M to C1 (*S. serrata*)	Feeding regimes	Individually	Round flat-bottomed plastic	None	100% MBD 100% *Artemia*	Until M metamorphosed to C1 or died	90 90		[39]
M to C1 (*S. serrata*)	Feeding regimes: Microbound diets (MBD)	Individually	Flat-bottomed circular aquaria (250 mL)	None	MBD (prepared using dried rotifers) MBD (prepared using *Artemia* meal) MBD (prepared using squid meal) MBD (prepared using fish meal) Live *Artemia*	Until M metamorphosed to C1 or died	46.67 46.67 60.00 60.00 80.00		[40]
M to C1 (*S. serrata*)	Feeding regimes: MBD prepared using squid meal (containing different levels of dietary cholesterol)	Individually	Flat-bottomed aquaria (250 mL)	None	MBD + 0.14% cholesterol level MBD + 0.20% cholesterol level MBD + 0.40% cholesterol level MBD + 0.80% cholesterol level MBD + 1.00% cholesterol level Live *Artemia*	Until M metamorphosed to C1or died	26.70 60.00 53.30 73.30 46.70 53.30		[41]
M to C1 (*S. serrata*)	Feeding regimes: MBD (containing various levels of supplemental dietary lecithin and cholesterol)	Individually	Flat-bottomed aquaria (250 mL)	None	MBD (containing lecithin 5.5 g.kg^−1^ + cholesterol 0.3 kg^−1^) MBD (containing lecithin 5.2 g.kg^−1^ + cholesterol 7.9 kg^−1^) MBD (containing lecithin 23.1 g.kg^−1^ + cholesterol 0.1 kg^−1^) MBD (containing lecithin 27.8 g.kg^−1^ + cholesterol 10.7 kg^−1^) MBD (containing lecithin 39.7 g.kg^−1^ + cholesterol 0.6 kg^−1^) MBD (containing lecithin 44.1 g.kg^−1^ + cholesterol 8.8 kg^−1^) Live *Artemia*	Until M metamorphosed to C1 or died	20 27 33 53 60 60 60		[42]
M to C1 (*S. serrata*)	Feeding regimes: MBD (containing various fish oil:corn oil ratios)	Individually	Flat-bottomed circular aquaria (250 mL)	None	MBD (fish oil:corn oil = 0:1) MBD (fish oil:corn oil = 1:2) MBD (fish oil:corn oil = 2:1) MBD (fish oil:corn oil = 3:1) MBD (fish oil:corn oil = 1:0) MBD (fish oil:corn oil = 1:1) Live *Artemia*	Until M metamorphosed to C1 or died	35 55 60 65 65 70 60		[43]
M to crablet stages (*S. paramamosain*)	Temp. 20 °C Temp. 24 °C Temp. 28 °C Temp. 32 °C Temp. 36 °C Temp. Ambient (27–30 °C)	Individually	Plastic cup with diameter of 6–9 cm	None	Frozen *Artemia* nauplii, frozen adult *Artemia* and artificial feed	45	0 86.67 96.67 80.00 0 93.33	0 3.74 4.50 3.95 0 4.38	[24]
M to C1 (*S. paramamosain)*	Feeding regimes	Individually	Plastics beakers (0.5 L)	None	Live *Acetes* (LA) Minced shrimp meat (MSM) Locally formulated feed (LFF) Commercial feed (CF) LA + MSM LA + LFF LA + CF	Until M metamorphosed to C1 or died	85–100		[44]
M to C1 (*S. paramamosain)*	Feeding regimes	Communal (250 ind./hole	Earthen dugout holes 60 × 60 × 20 cm (length × width × depth)		Live *Acetes* (LA) Minced shrimp meat (MSM) Locally formulated feed (LFF) Commercial feed (CF) LA + MSM LA + LFF LA + CF	10	47.9–87.5		[44]
C1 to several crablet stages (*S. paramamosain)*	Stocking density	110 ind/m^2^ 175 ind/m^2^ 230 ind/m^2^	PVC containers	Sand substrate	*Artemia* biomass and chopped peeled shrimp	15	71.3 61.7 57.5		[28]
C1 to several crablet stages (*S. paramamosain)*	Shelter	110 ind/m^2^	Cement tank	Clay brick Without clay brick	Peeled shrimp	17	25.3 13.5		[28]
C1 to several crablet stages (*S. paramamosain)*	Shelter types	100 ind/m^2^	Composite tank	Bricks Clamshell Without shelter	Peeled shrimp	21	40 41 30		[28]
Crablets of 7–10 days old (±C3) (*S. olivacea*)	Rearing medium	50 ind/m^2^	Fibre tank Hapa net	Paranet pieces	*Acetes* spp.	21	21.33 37.33		[31]
Z5 and M (mix) to crablet (day 7) (*S. olivacea*)	Rearing medium	5000 ind/tank 1500 ind/tank 1360 ind/tank	Circular fiberglass tank Rectangular cement tank Circular cement tank	None	Enriched *Artemia* nauplii	13	40.14 34.65 22.67		[32]
Z5 and M to C3 (*S. tranquebarica*)	Feeding regimes	Communally	Fiberglass tanks (vol. ± 4 tons)	Paranet pieces	Live *Artemia* nauplii + shrimp meat Live *Artemia* nauplii and artificial feed (shrimp post-larvae feed)	14	16.46 5.72		[20]
Z4–Z5 to C1 (*S. olivacea)*	Feeding regimes: substitution of nauplii *Artemia* (NA) with microdiet (MD)	12 ind./L	Conical fiberglass tank (filled with 150 L of seawater)		NA 100% NA 75% + MD 25% NA 50% + MD 50% NA 25% + MD 75% MD 100%	15	2.42 4.22 5.61 4.89 2.1		[45]

M = megalopa, C1 = crablet 1, SR = survival rate, SGR-_CW_ = specific growth rate in term of carapace width.

#### 3.2.1. Seed Stocking Strategies

The stocking density of 3 to 5 days old M in nursery tanks is typically around 1000–2000/ton of water [22,23]. The recommended stocking technique for M is based on the area (ind/m^2^) due to the fact that the M stage naturally stays at the bottom just before it moults to C1. Further studies related to the optimal density of the M per unit area need to be carried out in the future. The stocking of M with densities of 10, 20 and 30 ind/m^2^ in net cages within earthen ponds was shown to attain an average 50.5% survival rate [27] while the rearing of M in an aquarium with a density of 133 ind/m^2^ had a survival rate between 11.7–31.7% on different dietary treatments [19]. Beside the stocking density, another factor that needs to be considered in determining the stocking density of M is the optimal water depth that can be used during the maintenance process for water efficiency.

When C1 was cultured at high densities of 110, 175 and 230 crabs/m^2^ for 15 days, survival rates of 71%, 62% and 58% were reported, respectively [28]. A longer nursery period of 30 days with a lower density of 70 ind/m^2^ had a survival rate of 52–66% while a further extended nursery to 60 days with a density of 30 ind/m^2^ attained a survival rate of 64–67% [28]. A lower survival rate of between 21 and 37% was found by Syafaat et al. [30] for mud crab crablets (day 7–10) reared for 21 days at a density of 50 ind/m^2^. Hence, further research is needed to identify the factors controlling the survival rate in relation to the stocking density.

#### 3.2.2. Age of Megalopa

The age of M at moulting is likely to affect the moulting success of M to C1. Syafaat [24] reported a lower survival rate of 2–3 days old megalopa than for 5–6 days old during their moult from M to C1 (reared individually). In a communal culture condition, using hapa nets within an earthen pond of 80 cm water depth, older M (3 and 4 days) showed a better survival than Z5, 1 day old and 2 days old M [46]. Aside from cannibalism and other factors such as environmental conditions, feed nutrition and disease, the age of M at stocking is another factor that needs to be considered in order to maximise the survival from M to C1, particularly under an individual culture condition.

#### 3.2.3. Transportation of Megalopa Stage

During transportation, the avoidance of a stationary transport condition or continuous agitation has been indicated to reduce the probability of M to grasp each other [47]. The transportation of M is typically in plastic bags with a density of 200–300 ind/L [27]. The survival rate is usually lower at a higher density. For example, Quinitio and Parado-Estepa [47] reported that the survival of M over a 6 h simulated transport (including shaking) could reach 99.3 ± 1.6%, 93.0 ± 5% and 94.0 ± 3.8% for densities of 50, 100 and 150 ind/L, respectively, leading to a conclusion that a lower density, i.e., 50 ind/L, is preferable. Beside the density, the temperature also affects the survival rate of M during transportation. The survival during transport of M at a temperature of 28 °C was lower than at a temperature of 24 °C [47].

#### 3.2.4. Feeding Strategies

*Artemia* is often used as the main feed during the maintenance of M to crablet stages with additional dried shrimp, dried mud worms, prawn/shrimp meat, fish meat or artificial feed (shrimp larvae feed) being added as high as a 1–5 mg/L concentration or greater [16,19,45,48]. Chen et al. [49] clarified that M were able to capture prey whose sizes ranged from nauplii up to small adult *Artemia*. Apart from a suitable feed size, feed nutritional quality is another important component supporting the development of M into the C1 stage. The use of enriched *Artemia* (instar 2) resulted in a better survival than the use of only *Artemia* nauplii [16]. *Acetes* is a potential live food in a mud crab nursery from megalopa to the crablet stage. The rearing of megalopa on *Acetes* alone or combined with minced shrimp meat, locally formulated feed or commercial feed showed a better survival than treatments fed with minced shrimp meat, locally formulated feed or commercial feed alone [44]. It is recommended to start artificial feed supplementation at the M stage as the diet modulation of the gut evacuation time (GET) is expected to begin at this stage [50] while the digestive enzyme activity of mud crab larvae tends to increase with the increasing larval stage [51,52].

Supplementation with artificial feed (shrimp post-larvae feed) and *Artemia* showed a higher survival rate when compared with the treatment being fed *Artemia* alone in rearing from the Z5 and M stages [19,45]. The use of juiced artificial feed with added spirulina powder and digezym (containing amylase, protease, lactase and cellulose), together with *Artemia* nauplii, produced more C1 than the treatment being fed only artificial feed juice (without spirulina powder and digezym) and *Artemia* nauplii [53]. Shorter moulting intervals shown in M fed only with a microbound diet (MBD) or an MBD combined with *Artemia* suggested that an MBD can be formulated to contain beneficial nutrients insufficient in *Artemia* [39], which have been evaluated as eicosapentaenoic acid (EPA) and docosahexaenoic acid (DHA) [16,54,55] and a lipid profile [43].

The nursery culture of mud crab *Scylla* has always been heavily dependent on live prey, particularly *Artemia* [41,44]. Nonetheless, the nutritional profile of the live prey does not always meet the dietary requirement of the mud crab megalopae. A low content of both EPA and DHA in *Artemia* compromised the vitality of larvae [52,56,57]. An unsuitable lipid profile for marine crustaceans found in rotifer and *Artemia* nauplii could affect both the larval development and the survival of mud crab larvae [43]. MBD feed containing squid meal with proper cholesterol and lecithin levels provides the equivalent survival of M and can even be better than a diet of only live *Artemia* [40,41,42]. Furthermore, MBD feed containing a fish oil to corn oil ratio of as high as 1:1 showed a better survival of M compared with the treatment that was fed only *Artemia* nauplii [43] (Table 2).

As soon as M metamorphose to the crablet stage, they are fed with minced trash fish, green mussel, *Acetes* [38], fresh chopped shrimp or tilapia [28], brown mussel [58], frozen adult *Artemia* [24] or artificial feed (Table 2) [19,20].

## 4. Water Quality

Water quality conditions tolerable in both the larval rearing and the brood stock maintenance of mud crabs include a 28–35 ppt salinity (10–35 ppt for nursery and grow-out), a temperature of 27–32 °C, a pH of 7.5–8.5, a DO >4 ppm and ammonia <0.01 ppm [59]. Alkalinity >80 ppm has been suggested for mud crab farming, ideally at 120 ppm [60]. As the optimal water parameters for mud crab farming are still considered to be under development, the optimal water parameters for tiger prawns may be used as a guidance [60]. The optimum range of several important water quality parameters suggested for mud crab culture operation are shown in Table 3.

Temperature and salinity are two key parameters to be considered both in the larvae rearing and the nursery phases of mud crabs [24,30,33,61,62,63,64]. These two parameters greatly affect the physiological processes, having an impact on the growth of portunid crabs [65,66,67,68]. The recommended salinity for the rearing of M and C1 is between 20–25 ppt [30,33,61,62,63,64] while the recommended temperature is 28–30 °C [24,30,66]. The optimal temperature recommended for the moulting process of M to C1 is 28 °C with a lower temperature condition between 24 and 28 °C having been shown to be more conducive to the moulting process of M to C1; much preferred to a high temperature (i.e., up to 32 °C) or fluctuating temperature conditions [24].

The use of artificial feed during mud crab larvae rearing may trigger the growth of *Vibrio* spp. on the culture media [20]. Hence, the use of antibiotics (which are still globally restricted) to control the growth of *Vibrio* spp. is still needed. However, the rearing of portunid crab larvae with probiotics has been proven to improve the production of mud crab crablets [24] and they are able to suppress and control the development of pathogenic *Vibrio* spp. populations [3,69] whereby their use is recommended over the use of antibiotics.

**Table 3:** Water quality parameters suggested for a mud crab nursery and grow-out culture operation.

Parameters	Optimum Range/Value	Sampling Frequency
Dissolved oxygen (DO)	>5 ppm (mud crabs are tolerant of low oxygen levels) [60]	Twice a day
pH	7.5–8.5(<0.5 daily variation) [59]	Twice a day
Temperature	28–30 °C [24,30,66,68]	Daily
Salinity	20–25 ppt [30,33,61,62,63,64]	Daily
Total ammonia nitrogen (TAN)	<3 ppm (crablets have a tolerance to high ammonia) [60]	Weekly
Un-ionised ammonia (NH_3_)	<0.01 ppm [59]	Weekly
Nitrite (NO_2_)	<10 ppm at salinity >15 ppt; <5 ppm at salinity <15 ppt [60]	Weekly
Alkalinity	>80 ppm (ideally 120 ppm) [60]	Weekly
Hardness	>2000 ppm [60]	Weekly
Hydrogensulphide	<0.1 ppm [60]	Weekly
Turbidity	20–30 cm [60]	Daily

## 5. Harvest and Transportation of Crablets

The harvesting process for the nursery phase (M to crablets or from early crablets (C1–C2) to a larger crablet phase) occurs between two and four weeks after stocking. Long nursery periods in small areas (high density with minimal shelter) will lead to a high mortality due to cannibalism. Two methods, the dry (moist) method [12,70] and the wet method (with water) [71], can be used to deliver harvested crablets to grow-out farms (Table 4). The dry method of transportation, without water, uses materials that function as a shelter within containers. The wet method provides water, typically in a plastic bag, supplied with sufficient oxygen above the water. Shelter is also a requirement of the wet method of transportation, i.e., a nylon net.

## 6. Sex Differentiation in the Crablet Stage

**Figure 2:**
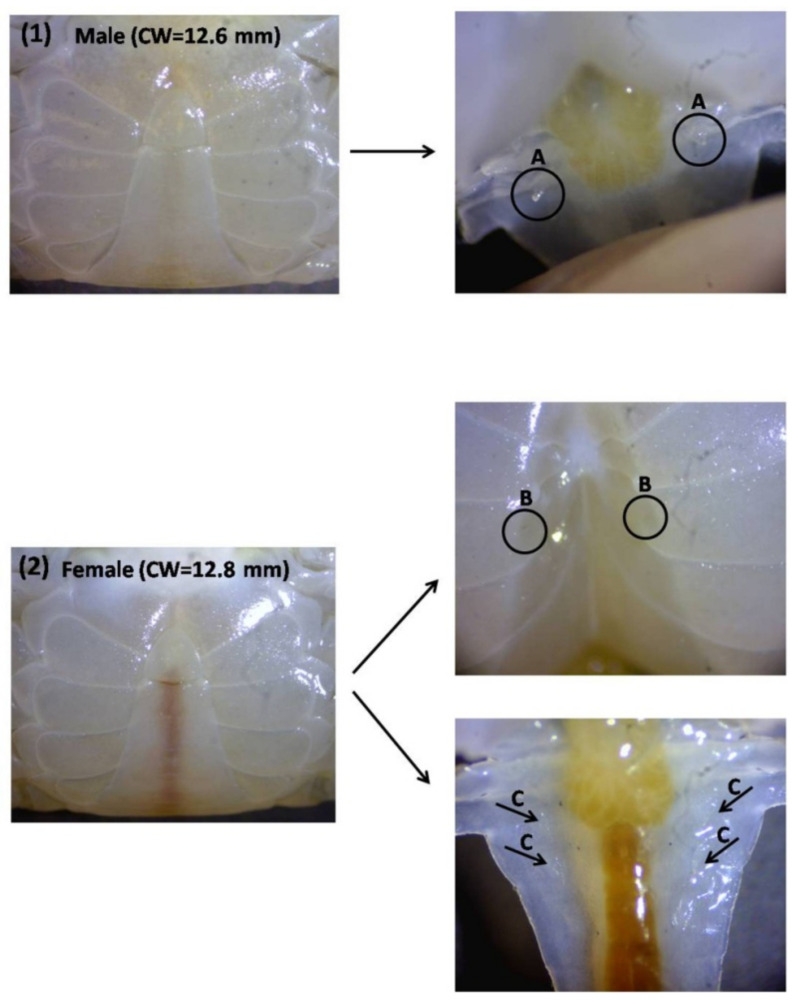
The appearance of the abdominal flap of mud crab crablets *S. paramamosain* (male and female). (**A**) gonopod (copulatory pleopods), (**B**) gonopore and (**C**) biramous pleopods.

The morphological sex differentiation of mud crabs is mainly based on the shape of the abdominal flap and body size dimensions [72,73]. In the earlier crablet stages of *S. paramamosain*, the difference of the abdominal flap can be seen clearly at the C5 stage (CW of ±1 cm) using a microscope (magnification of 8–20×) whereas in the ±C9 stage (CW of ±2 cm), the difference can be seen with the naked eye [25]. In mud crab *S. serrata*, the different shape of the male and female abdomen enables the sexing of crabs above a 3 cm carapace width with a casual inspection while below this size, the sex can be determined at 30× by means of a binocular microscope [74]. Females are recognised by the presence of four pairs of biramous pleopods and an oviduct depression (gonopore) on the sternites of the sixththoracic segment while males are recognised by the presence of copulatory pleopods and the absence of oviduct depressions [74] (Figure 2).

The ability to distinguish the sex of mud crabs in the crablet phase can be an added value for mud crab hatcheries, allowing a sale at a higher price. In a mud crab culture, a monosex culture is highly profitable as mud crabs show an obvious sexual dimorphism. A monosex culture of all males shows higher specific growth rates (SGRs) compared with all females [9] and a culture trial of monosex cultures (all male and all female) has yielded a significantly higher production and survival compared with a mixed culture [75]. Therefore, the availability of monosex seeds is also important to support monosex mud crab farming in brackishwater ponds. A further study on sex reversal technology for mud crabs to produce monosex crab seeds is important to be conducted in the future.

## 7. Conclusions and Recommendations

A mud crab nursery in the hatchery beginning from the M stage is important for production because the stocking of M directly to ponds has not shown good results when compared with species such as *P. pelagicus*. Although operating a mud crab nursery for crablets individually exhibits a higher survival when compared with communal rearing, the individual nursery involves a higher complexity and resourcing so that it is necessary to consider rearing technology using automatic machinery especially for the feeding process. A mud crab nursery can be carried out both indoors (using plastic, fiberglass or concrete tanks) and outdoors (earth, lined or concrete ponds) within net cages. In all applications, the rearing needs to be accompanied by the use of adequate shelter, particularly for a communal nursery.

The availability of adequate food mainly contributes to suppressing cannibalism. Feed in the form of live *Artemia* is still the most common main feed in the M phase but *Artemia* combined with additional feed (artificial feed, worm meal, shrimp meat) provides a better survival compared with *Artemia* alone. An MBD of the right composition is a feed candidate to replace or substitute for *Artemia* in the M phase.

Although mud crabs are believed to show a good resilience in nature and survive in extreme conditions (e.g., in mud and without water), the water quality parameters for a shrimp culture can be used as a guide in mud crab farming activities. Further studies related to the transportation of crablets need to be carried out to produce practical, inexpensive methods and to determine the extent of crablet resistance during transportation without water. In addition, an improved knowledge of the sex differentiation of mud crabs during the early crablet stage would be expected to provide added value to mud crab hatcheries.

Various aspects of a mud crab hatchery described in this paper are expected to facilitate practitioners in understanding the natural conditions of mud crabs, genus *Scylla*, and implementing them in nursery operations. It is clear that current hatchery practices have room for development and the farming industry could improve in several aspects with future research on exploring the feasibility of individual rearing and biofloc application in controlling water quality, establishing monosex seed production as well as improving methods of transportation during critical mud crab stages. This paper is also expected to provide guidelines to researchers in conducting future research and development on a mud crab nursery, subsequently transferring the fundamental knowledge to the farming community.

## Figures and Tables

**Table 1:** Moulting interval and carapace sizes of mud crabs, *S. paramamosain*, in the early crablet stages (at an ambient temperature of 27–30 °C).

Crablet Stage	Moulting Interval (Days) ± SD	Carapace Width (mm) ± SD	Carapace Length (mm) ± SD
C1 to C2	4.67 ± 0.50	3.41 ± 0.10 (C1) 4.80 ± 0.34 (C2)	3.03 ± 0.10 (C1) 3.71 ± 0.32 (C2)
C2 to C3	5.88 ± 0.99	6.00 ± 0.25 (C3)	4.55 ± 0.30 (C3)
C3 to C4	7.34 ± 1.03	7.83 ± 0.61 (C4)	5.69 ± 0.40 (C4)
C4 to C5	9.33 ± 2.18	9.56 ± 0.73 (C5)	6.78 ± 0.46 (C5)
C5 to C6	11.95 ± 2.14	12.08 ± 1.07 (C6)	8.49 ± 0.69 (C6)

SD = standard deviation.

**Table 4:** Literature related to the transportation of mud crab crablets.

Life Stage of Mud Crabs (Days/Sizes)	Crablet (D20) *S. paramamosain*	Crablet (With Width Carapace Less Than 1 cm) *S. paramamosain*	Crablet (D37) *S. tranquebarica*
Methods	Wet	Dry (without water)	Wet
Medium	Plastic bags (filled with oxygen)	Plastic bags (filled with oxygen)	Styrofoam box (40 × 50 cm)
Density	50, 100, 150 ind/pack	200 and 300 crablets	700 ind/box
Shelter	A nylon net (20 × 40 cm)	Wet cloth along with seaweed (*Gracillaria* spp.)	Paranet
Duration (h)	5	5	6
Survival rate (%)	88–97	98–99	>95
Reference	[71]	[70]	[12]

## Data Availability

Not applicable.
